# Five days of inpatient scoliosis-specific exercises improve preoperative spinal flexibility and facilitate curve correction of patients with rigid idiopathic scoliosis

**DOI:** 10.1007/s43390-024-00965-1

**Published:** 2024-09-26

**Authors:** Yunli Fan, Michael K. T. To, Guan-Ming Kuang, Nan Lou, Feng Zhu, Huiren Tao, Guangshuo Li, Eric H. K. Yeung, Kenneth M. C. Cheung, Jason P. Y. Cheung

**Affiliations:** 1 Department of Orthopaedics, Guangdong, The University of Hong Kong – Shenzhen Hospital, Shenzhen, Guangdong People’s Republic of China; 2 Department of Orthopaedics and Traumatology, The University of Hong Kong, Hong Kong SAR, China; 3 Department of Physiotherapy, Department of Orthopaedics and Traumatology, Guangdong, The University of Hong Kong – Shenzhen Hospital, Shenzhen, People’s Republic of China

**Keywords:** Adolescent idiopathic scoliosis, Scoliosis-specific exercise, Preoperative spine flexibility, Fulcrum-bending

## Abstract

Preoperative spine flexibility plays a key role in the intraoperative treatment course of severe scoliosis. In this cohort study, we examined the effects of 5 day inpatient scoliosis-specific exercise (SSE) on the spinal flexibility of patients with adolescent idiopathic scoliosis before surgery. A total of 65 patients were analyzed. These patients were divided into a prospective cohort (*n* = 43, age: 15 ± 1.6 years, 36 girls and 7 boys, Lenke class 1 and 2, Cobb angle: 64 ± 11°) who underwent spinal fusion in 2020, and a retrospective cohort (*n* = 22, age: 15 ± 1.5 years, 17 girls and 5 boys, Lenke class 1 or 2, Cobb angle: 63 ± 10°), who underwent surgery between 2018 and 2019 and did not receive preoperative SSE. Rigid scoliosis was defined as a reduction of less than 50% in Cobb angle between the preoperative fulcrum bending and initial standing curve magnitude. In the prospective cohort, 21 patients (Cobb angle: 65 ± 11°) presented with rigid thoracic scoliosis (pre-SSE fulcrum bending: 40 ± 9°, 39% reduction), and therefore received 5-day SSE to improve their preoperative spinal flexibility (SSE group), whereas 22 patients (Cobb angle: 63 ± 12°) presented with flexible thoracic scoliosis (pre-SSE fulcrum bending: 27 ± 8°, 58% reduction), and therefore underwent surgery without preoperative SSE (non-SSE group). For patients who received 5-day preoperative SSE for 4 h every day, the International Schroth Three-Dimensional Scoliosis Therapy technique was implemented with an inpatient model. After 5 days of SSE, improvements in Cobb angle with post-SSE fulcrum-bending radiography (23 ± 7°, 66% reduction) and pulmonary function (forced expiratory volume in 1 s/forced expiratory volume: 87% before SSE and 92% after SSE, *p* < 0.01) were observed. At the postoperative day 5, the degree of scoliosis had reduced from 44 ± 6.6° to 22 ± 6° in the SSE group, which is 1° less than the Cobb angle obtained on post-SSE fulcrum-bending radiography. In the non-SSE group, the degree of scoliosis decreased to 26 ± 5.7°. In the retrospective cohort, the degree of scoliosis decreased to 35 ± 5°, with the group also having higher postoperative pain (Visual Analog Scale score = 7, range = 5–10) and an extended hospitalization duration (11 ± 3 days). At 2-year follow-up, curve correction was found to be maintained without adding-on or proximal junctional kyphosis. Compared with the non-SSE group, the SSE group exhibited a greater curve correction (66%) with a shorter hospitalization duration (5 ± 1 days) and a lower degree of postoperative pain (Visual Analog Scale score = 4, range = 3–8). Taken together, our findings indicate that 5 day SSE improves preoperative spinal flexibility and facilitates curve correction.

## Introduction

Segmental spinal instrumentation is performed in patients with a scoliotic curvature of 50° or greater to prevent the progression of scoliosis [1]. Progressive scoliosis may lead to cardiovascular or respiratory dysfunction [1–3]. Operative correction improves postoperative truncal balance and cosmesis [4], and low correction rates may result in poor postoperative truncal balance and increase the risk of adding-on curve and junctional proximal kyphosis [4]. In addition, rigid curves are difficult to correct and require osteotomy to achieve optimal intraoperative correction, which increases the risk of postoperative neurological injuries [5]. Spinal flexibility determines the intraoperative correction, and preoperative fulcrum-bending radiography predicts the postoperative correction [6]. Therefore, in patients with stiff scoliosis, stretching exercises may aid in improving their flexibility and mitigating the risk of correction. Overall, preoperative exercise is an effective risk-free technique for improving surgical outcomes.

Scoliosis-specific exercise (SSE) is a popular intervention that is easily administered as either an inpatient or an outpatient model [7]^.^ This intervention reduces Cobb angles in patients with mild to moderate idiopathic scoliosis while mitigating the risk of progression [7–13]. Despite its positive effects, the efficacy of SSE in treating patients with severe scoliosis during the preoperative stage remains unclear. SSE relies on active spine elongation in the longitudinal direction, as well as diagonal stretching coupled with rotational breathing, to mobilize the deformed rib cage and expand the curve’s concavity [14]. Therefore, SSE is regarded as a potential strategy for improving spinal flexibility.

In a previous study, Yang et al. [15] reported a reduced hospitalization duration and accelerated postoperative recovery in patients who were treated using an enhanced recovery after surgery (ERAS) protocol. In another study, Julien-Marsollier et al. [16] adopted standardized multimodal analgesia and bedside exercises to control pain and enhance postoperative recovery. To the best of our knowledge, no studies have examined the clinical utility of preoperative exercise in ERAS. Achieving greater preoperative spinal flexibility can enable a greater degree of intraoperative correction and less postoperative paraspinal muscle tenderness [6]. Taken together, these findings confirm the potential utility of SSE in improving spinal flexibility, maximizing curve correction, and relieving postoperative pain. In this study, we developed a novel structured SSE program to improve the preoperative spinal flexibility of patients with stiff scoliosis. We hypothesized that 5-day preoperative SSE can improve the degree of spinal flexibility and thereby facilitate curve correction.

## Materials and methods

This study was conducted in accordance with the principles of the Declaration of Helsinki. Ethical approval ([2019]253) was obtained from the review board of The University of Hong Kong—Shenzhen Hospital. Written informed consent to publish the following data was obtained from the patients or their parents or legal guardians before the study.

### Patients and outcome measurements

Patients with thoracic spinal scoliosis who underwent spinal fusion in 2020 were included in the prospective cohort, and patients who underwent surgery between 2018 and 2019 were included in the retrospective cohort. Our operative protocol comprised Schwab grade 1 osteotomy (only inferior articular process excision) at apical vertebrae, cantilever bending correction, and instrumented fusion. Patients meeting the following criteria were included for analysis: being 10– 17 years of age, having thoracic spinal scoliosis (Lenke class 1 or 2), and having a Cobb angle of 50° or greater with thoracic scoliosis. Patients meeting the following criteria were excluded from the study: having any diseases other than adolescent idiopathic scoliosis (AIS), having any disabilities that may hinder exercise, and having non-thoracic spinal scoliosis. Before surgery, standing whole-spine posteroanterior radiographs were obtained to evaluate each patient’s curve magnitude. Fulcrum-bending radiographs were also obtained to predict the postoperative correction course. Fulcrum-bending radiography was performed by a trained radiologist to prevent systematic errors that typically arise from patient positioning. Measurements were conducted with each patient lying on the convex side of their thoracic scoliosis (right side of the thoracic spine), with hard triangular prism-shaped foam placed underneath the ribs at the apical vertebral level to lift the ipsilateral shoulder off the X-ray table for maximum passive bending force on the curve [6]. This foam was rounded with padded edges, with each face representing a different height (17.0, 17.5, and 21.0 cm) of the fulcrum-bending foam [17]. This design was intended to passively expand the curve’s concavity to evaluate spinal flexibility. In patients with AIS, fulcrum-bending correction strongly correlates with postoperative curve correction (with a fulcrum-bending correction index of 100%) for proximal and main thoracic curves [18]. Exercise presumably affects the flexibility of not only the thoracic spine but also the lumbar spine. Given the flexibility of the lumbar and thoracolumbar spine, in this study, patients with thoracolumbar or lumbar spinal scoliosis were excluded to control study heterogeneity. In patients with thoracic spinal scoliosis, any change in thoracolumbar curve flexibility after SSE may compensate for changes in thoracic curve magnitude on fulcrum bending. Therefore, in this study, fulcrum-bending Cobb angles were measured only from the upper end vertebrae to T11, whereas the thoracolumbar segment of T12-L1 was not measured to avoid measurement errors. Cobb angles and spinal flexibility were independently measured by a radiologist and a spine surgeon who were blind to the study objectives. Spinal flexibility was determined as the difference between Cobb angles measured with standing and fulcrum-bending radiography (spinal flexibility = $\frac{\text{initial standing Cobb angle }-\text{ fulcrum}-\text{bending Cobb angle}}{\text{initial standing Cobb angle}})$. Rigid scoliosis was defined as a reduction of less than 50% in Cobb angles between the pre-SSE fulcrum-bending curve magnitude and initial curve magnitude [19]. Patients with rigid scoliosis received 5-day preoperative inpatient SSE to improve their spinal flexibility. Changes in spinal flexibility were calculated as the difference between pre-SSE and post-SSE spinal flexibility.

Pulmonary function was routinely evaluated in a respiratory laboratory by an operator who was blind to our SSE program. Forced vital capacity (FVC, L) and forced expiratory volume in 1 s (FEV_1_, L) were measured three times using a spirometer (Sensormedics 2000, Yorba Linda, CA, USA) with the patients in an upright position. Calibration and pulmonary function tests were conducted in accordance with the guidelines of the American Thoracic Society [20]. The absolute values of FVC and FEV_1_, with a percentage of the predicted values of FEV_1_/FVC, were determined. The curve magnitude with different body heights was statistically corrected to determine the percentage of the predictive values of FEV_1_/FVC.

Treatment satisfaction (satisfaction domain of the Scoliosis Research Society-22 Patient Questionnaire [SRS-22r]) [21], pain (Visual Analog Scale [VAS] score = 0–10), postoperative complications, and hospitalization duration were documented after surgery [22].

### Preoperative 5-day SSE protocol

Our SSE program involved the following. First, active spine-elongating exercises were performed in the sitting and bow standing positions (Table 1: a, b) to symmetrically elongate the spine in the longitudinal direction. Second, asymmetrical corrective exercises were performed with diagonal stretching by pulling the shoulder blade on the concave side in the cranial direction, with the pelvis on the convex side in the caudal direction (Table 1: c, d), to enhance the stretching of the curve’s concavity in the diagonal direction. Third, core exercises were performed to strengthen the paraspinal and abdominal muscles (Table 1 e, f). Fourth, exercises were conducted in a side-lying position (Table 1: g), with a sling anchored on the apical prominence of the curve’s convexity (convex side down), to passively expand the curve’s concavity with suspension. During this exercise, active stabilization of the pelvis was required. All corrective exercises were performed while rotational breathing was practiced (Table 1: h). The patients were instructed to deeply inhale in an angular direction to expand the curve’s concavity on the back and the flat zone on the anterior trunk to enable the deformed rib cage to self-mobilize (Table 1: h). A total of 50 breathing repetitions were performed for each set of corrective exercises, with each session comprising 5 sets, with 5 sessions of each exercise conducted daily, amounting to 4 h of SSE every day for a total of 5 days. Before surgery, the patients were trained to adopt the Schroth corrective posture during standing and sitting [7, 14].

**Table 1:** Details of the 5-day inpatient SSE program

Exercise	Practical principle
Hanging exercises	**a.** Hanging exercise in sitting. This stretches the spine in a longitudinal direction **b.** Hanging exercise in bow standing. This produces a longitudinal lengthening in the spine and stretches posterior muscles of the lower limbs. Patients must elongate the spine in a caudal to cranial direction with exhalation
Asymmetrical corrective exercises	**c.** Corrective exercise alongside rotational breathing in kneeling to stretch the curve concavity **d.** Corrective exercise alongside rotational breathing prone to stretch the curve concavity
Core muscle training exercises	**e.** Plank exercise to strengthen abdominal muscles **f.** Arched-back exercise to strengthen back muscles
Corrective exercise with passive stretching	**g.** Corrective exercise with hanging assistance to expand the curve concavity with a sling anchored the apical prominence of the curve convexity
Rotational breathing technique	**h.** An axial cut of computed tomography scan showing the curve concavity in the posterior back and the flat zone in the anterior ribcage. These areas require expansion with inhalation and eccentric contraction with exhalation. Rotational inhalation is performed to expand the curve concavity in backward, sideward, and upward directions

Preoperative SSE was administered with a 1-on-1 inpatient model. The patients were admitted 5 days before surgery to perform 4 h of SSE daily in addition to the scheduled preoperative assessments. The SSE program was scheduled from 9:30 to 11:30 AM and from 3:30 to 5:30 PM every day. Two physiotherapists licensed in the International Schroth Three-Dimensional Scoliosis Therapy approach, with 10 years or more of experience in treating scoliosis, conducted the SSE sessions. Their techniques were focused on rotational breathing, spinal elongation, and overcorrection with sling assistance to expand the curve’s concavity. Before each treatment session, the patients received information on the objective, dosage, and daily schedule of the SSE program to ensure enhanced cooperation with the physiotherapists. Consequently, both the physiotherapists and the patients were aware of the SSE program details.

In addition to the 5 day SSE program, the patients underwent routine preoperative assessments. These assessments included X-ray radiography (standing and fulcrum bending) to evaluate curve flexibility and identify touch vertebrae (operative strategy-making). Computed tomography scans were performed to measure pedicle diameter and evaluate vertebral rational deformity. Magnetic resonance imaging was conducted if a neurological symptom or atypical curve pattern was observed before surgery. Cardiopulmonary function assessments were conducted to evaluate the risks associated with anesthesia and determine potential postoperative complications. In addition, perioperative physiotherapy care, including walking and breathing exercises, was provided to patients undergoing spinal surgery at our center. This care was initiated to enhance recovery after surgery. The patients began walking and postural training on day 2 after surgery if no complications occurred.

### Statistical analysis

A pilot study involving patients with rigid scoliosis was conducted to determine the effectiveness of our 5-day inpatient SSE program in reducing the magnitude of the fulcrum-bending curve. The results indicated a reduction of 12° in Cobb angles with fulcrum bending after SSE. In a previous study on SSE with an effect size of 1.7, Fan et al. [8] reported a reduction of 5.5° in Cobb angles measured in a standing position after SSE, whereas the reduction was only 2° in the control group. In the current study, considering a type I error of 5% and a power of 95%, 11 patients per group were required for the proposed 2‐tailed test. To account for potential dropouts, the sample size was increased by 1.5 times, resulting in a requirement of 17 patients per group.

Descriptive statistics were calculated for baseline variables, including demographics, Cobb angle, pulmonary function (FVC and FEV_1_), sport intensity (self-reported hours per day over the preceding 6 months), treatment history (self-reported bracing [yes or no with hours/day if yes], SSE [regular SSE: hours/day; non-regular SSE: yes/no]), VAS score (0–10) and SRS-22r score (satisfaction domain). Continuous variables are presented as means ± standard deviations, with or without median values, depending on the results of normality tests. The primary outcome of this study was the change in fulcrum-bending Cobb angles after SSE and surgical correction. Mixed-model analysis of variance was conducted if the normality assumptions for Cobb angles were met, whereas a nonparametric Friedman-type test was conducted if these assumptions were violated. Intergroup comparison of Cobb angles was conducted at baseline, before and after fulcrum-bending SSE, on postoperative day 5, at 6-month follow-up, and at 2-year follow-up. Post hoc pairwise comparisons were conducted with repeated measures and Bonferroni adjustments if significant differences were detected in the intragroup or intergroup comparisons. All statistical analyses were conducted using IBM SPSS Statistics version 27.0 (IBM, Armonk, NY, USA).

## Results

### Preoperative spinal flexibility

**Fig. 1:**
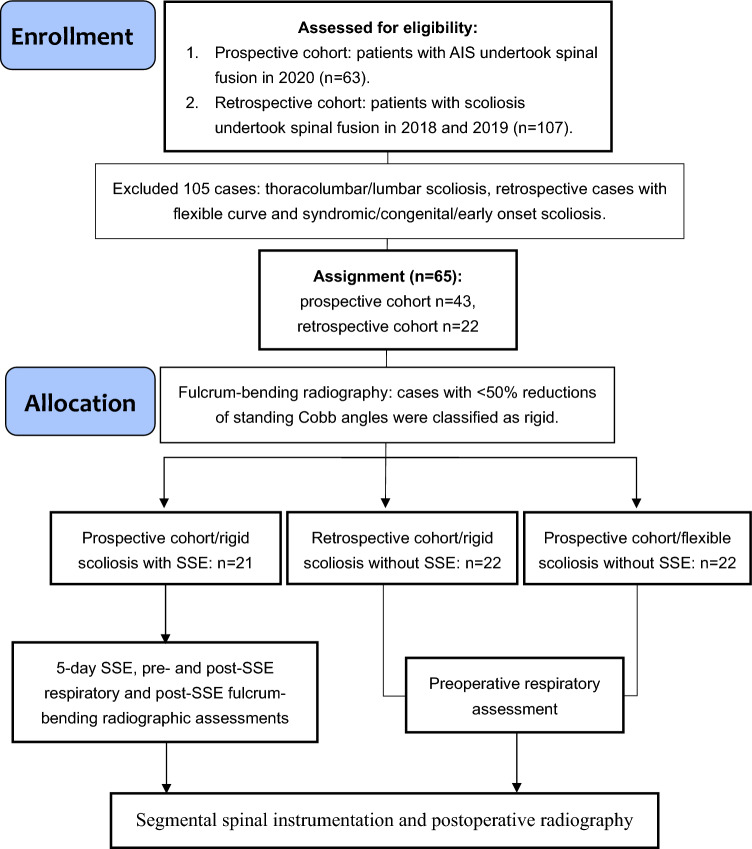
Study flow chart. *AIS*: adolescent idiopathic scoliosis. *SSE*: scoliosis-specific exercise

After screening for eligibility, we examined 43 patients and analyzed 22 historical cases (age: 15 ± 1.6 years, 36 girls and 7 boys, Cobb angle with major thoracic scoliosis: 64 ± 11°, Fig. 1). A total of 21 patients experienced a reduction of 26 ± 6.6° in Cobb angle (pre-SSE Cobb angle in a standing position: 65 ± 11°, pre-SSE Cobb angle with fulcrum bending: 40 ± 9°, spinal flexibility: 39%) and were classified as rigid (Table 2). According to a previous study, a difference of 1° to 6° exists between fulcrum-bending radiography and postoperative correction [23]. Given this level of perioperative flexibility, the predicted postoperative correction in this study ranged from 36 ± 6.1° (residual Cobb angle with maximal correction) to 58 ± 5.7° (residual Cobb angle with minimal correction). In addition, assessments of pre-SSE pulmonary function revealed relatively low FVC and FEV_1_ values (Table 2). Consequently, these patients received 5-day SSE preoperatively to improve their spinal flexibility and pulmonary function (SSE group). A total of 22 patients exhibited a 36° reduction in Cobb angle (pre-SSE Cobb angle in a standing position: 63 ± 12°, pre-SSE Cobb angle with fulcrum bending: 27 ± 8°, spinal flexibility: 58%) and were classified as flexible; hence, no SSE was initiated preoperatively (Table 2). Patients in the retrospective cohort who did not receive SSE exhibited a 24° reduction in Cobb angle (Cobb angle in a standing position: 63 ± 10°, Cobb angle with fulcrum bending: 39 ± 8°, spinal flexibility: 39%) and were classified as rigid. The three groups exhibited no differences in age, sex, body mass index, sport intensity, medical history, curve pattern, or initial standing curve magnitude at baseline (Table 2). The levels of preoperative pain are listed in Table 2.

**Table 2:** Demographic and clinical characteristics of patients

	Rigid scoliosis with SSE	Rigid scoliosis without SSE	Flexible scoliosis without SSE	*P*
Age: mean ± SD	15 ± 1.6	15 ± 1.5	15 ± 1.5	1
Sex: n (female / male)	17 / 4	17/5	19 / 3	0.7
Body mass index	22 ± 1.3	21 ± 1.2	22 ± 1.7	0.2
Sport intensity				
Hours/day; mean ± SD	1.3 ± 0.5	1.1 ± 0.6	1.0 ± 0.5	0.2
Previous treatment history: n				
No treatment Brace Brace with SSE SSE	12 7 2 0	14 6 2 0	9 11 2 0	0.6
Curve magnitude: mean ± SD				
Preoperative: pre-SSE: standing fulcrum bending post-SSE: fulcrum bending Postoperative: Day 5/standing 6-months/standing 2-years/standing	65 ± 11° 40 ± 9° 23 ± 7°^**δ**^ 22 ± 6° 21 ± 7° 23 ± 6°	63 ± 10° 39 ± 8° – 35 ± 5° 34 ± 5° 34 ± 4°	63 ± 12° 27 ± 8° – 27 ± 6° 26 ± 6° 29 ± 6°	0.8 < 0.01 < 0.01 < 0.01 < 0.01 < 0.01
Curve pattern: n				
T4 to T11-12 T4 to L1-2 T5 to T11-12 T5 to L1-2 T6 to L1-2	1 1 11 8 -	4 1 6 8 3	2 1 8 8 3	0.7
Respiratory function				
Pre-SSE: FVC FEV1 FEV1/FVC Post-SSE: FVC FEV1 FEV1/F VC	2.5 ± 0.4 2.2 ± 0.3 87% 2.7 ± 0.5^**δ**^ 2.5 ± 0.4^**δ**^ 92%^**δ**^	2.6 ± 0.3 2.3 ± 0.3 88% – – –	2.7 ± 0.4 2.5 ± 0.4 92% – – –	0.2 0.02 < 0.01
Pain score:				
median (range) Preop VAS Postop VAS	0 (0–5) 4 (3–8)^**δ**^	0 (0–3) 7 (5–10)^**δ**^	0 (0–5) 5 (4–8)^**δ**^	0.9 < 0.01
Postop complication: n				
Tension pneumothorax	0	1	0	–
Postop hospitalization days:				
mean ± SD	5 ± 1	11 ± 3	5 ± 1	< 0.01
Treatment satisfaction				
mean ± SD	4.8 ± 0.3	4.2 ± 0.5	4.4 ± 0.4	< 0.01

*F* female. *M* male. *T* thoracic. *L* lumbar. *SSE* scoliosis-specific exercise. *Postop* postoperative. *FVC* forced vital capacity. *FEV1* forced expiratory volume in 1 s, *SSE* scoliosis-specified exercise. *VAS* Visual Analog Scale

^δ^ Significant within-group difference

Overall, the SSE group demonstrated strong adherence to the SSE program, effectively performing all prescribed supervised exercises. No discomfort was reported during or after the 5-day inpatient SSE program. After the SSE program, the SSE group exhibited greater curve corrections with fulcrum bending compared with the non-SSE rigid and non-SSE flexible groups (Table 2). Post-SSE fulcrum-bending radiographs revealed Cobb angles of 23 ± 7.3° at T4/5 to T11, indicating a 66% reduction and an additional improvement of 16° compared with the pre-SSE fulcrum-bending radiographs of the SSE group. Given this level of preoperative flexibility, the predicted range of postoperative correction was between 18° and 30°, reflecting median values with minimal and maximal correction [24]. Post hoc power analysis revealed a 95% statistical power for this study regarding changes in preoperative spinal flexibility after SSE.

Low FEV_1_ and FEV_1_/FVC values were observed in patients with stiff scoliosis, particularly in the SSE group and the retrospective cohort without SSE (Table 2). After the 5-day inpatient SSE program, the values of FEV_1_ and FEV_1_/FVC significantly increased in the SSE group (Table 2).

### Surgical procedure and postoperative results

The patients underwent inferior articular process excision at apex and posterior instrumented fusion at various spinal levels: from T4/5 to L1-3 (SSE: *n* = 11, non-SSE: *n* = 23), from T4/5 to T11/12 (SSE: *n* = 3, non-SSE: *n* = 9), from T2 to L1-3 (SSE: *n* = 3, non-SSE: *n* = 7), and from T5 to L4 (SSE: *n* = 3, non-SSE: *n* = 3). One prospective and two retrospective cases with rigid scoliosis (T4/5 to L1/2) underwent Ponte osteotomy in addition to pedicle screw constructs. A mixed model with repeated measurements revealed a significant group effect (repeated analysis with pre-SSE fulcrum bending: *F* = 7.7, *P* = 0.001; repeated analysis with post-SSE fulcrum bending: *F* = 13.3, *P* < 0.001) and time × group effect (repeated analysis with pre-SSE fulcrum bending: *F* = 38.6, *P* < 0.001; repeated analysis with post-SSE fulcrum bending: *F* = 26.8, *P* < 0.001) across 5 testing time points (at baseline, before and after fulcrum-bending SSE, on postoperative day 5, at 6-month follow-up, and at 2-year follow-up). Surgical technique, namely Ponte osteotomy performed (*n* = 3) at the whole curve, had no statistical effects in the postoperative Cobb angle of this study cohort. Post hoc analysis identified significant intergroup differences in fulcrum bending and postoperative correction between groups (Table 2). On postoperative day 5, the average scoliosis Cobb angle for all patients had decreased by 40 ± 7.3° to a residual 24 ± 6.4°, and this reduction was maintained at the 6-month and 2-year follow-up. Compared with the non-SSE group, the SSE group achieved greater postoperative correction (Table 2), with a Cobb angle 1° less than the post-SSE fulcrum-bending measurement for 21 patients with stiff scoliosis. These findings confirmed the reliability of preoperative fulcrum-bending radiography in predicting surgical curve correction [19], with a Cobb angle falling within the measurement error range. This Cobb angle was 5° less than that of the 22 patients without SSE and 13° less than that of the 22 retrospective patients without SSE. According to our SRS-22r results, increased treatment satisfaction, reduced postoperative pain, and reduced hospitalization duration were reported for patients who received 5-day SSE preoperatively (Table 2). No instances of scoliosis deterioration or adding-on or proximal junctional kyphosis were detected at the 6-month or 2-year follow-up.

## Discussion

Spinal flexibility is routinely evaluated before surgery to predict the correctability of scoliosis deformities [19]. Preoperative flexibility facilitates extensive operative correction and mitigates the risk of postoperative complications. Preoperative fulcrum-bending radiography is a valid technique for predicting surgical correction outcomes [6]. To enhance the effects of surgical correction, halo-gravity traction is used before surgery to increase the patient’s spinal flexibility, particularly in cases of congenital and syndromic scoliosis with rigid curves [25]. This technique involves the use of skull pins to apply a constant force that elongates the spine [25]. Despite its positive effects, this technique requires an extended hospital stay and is associated with risks such as infection at the pin sites and potential pain in the cervicothoracic area [26]^.^ In addition, halo-gravity traction is rarely indicated for patients with AIS. To the best of our knowledge, no structured non-operative program has yet been developed to improve the spinal flexibility of patients with AIS before surgery.

SSE is an alternative non-operative approach that elongates the spine in the longitudinal direction, stretches the curve’s concavity in the diagonal direction, mobilizes the deformed rib cage in the angular direction (aided by deep rotational breathing patterns), and symmetrically strengthens the paraspinal muscles [14]. This approach has potential in improving preoperative spinal flexibility. Despite its effectiveness, no guidelines have yet been established for the use of SSE preoperatively to improve spinal flexibility. Given that spinal elongation is a fundamental aspect of SSE, its effect on spinal flexibility warrants further investigation. To our knowledge, this is the first study to examine the clinical utility of perioperative SSE in patients with stiff scoliosis.

**Fig. 2:**
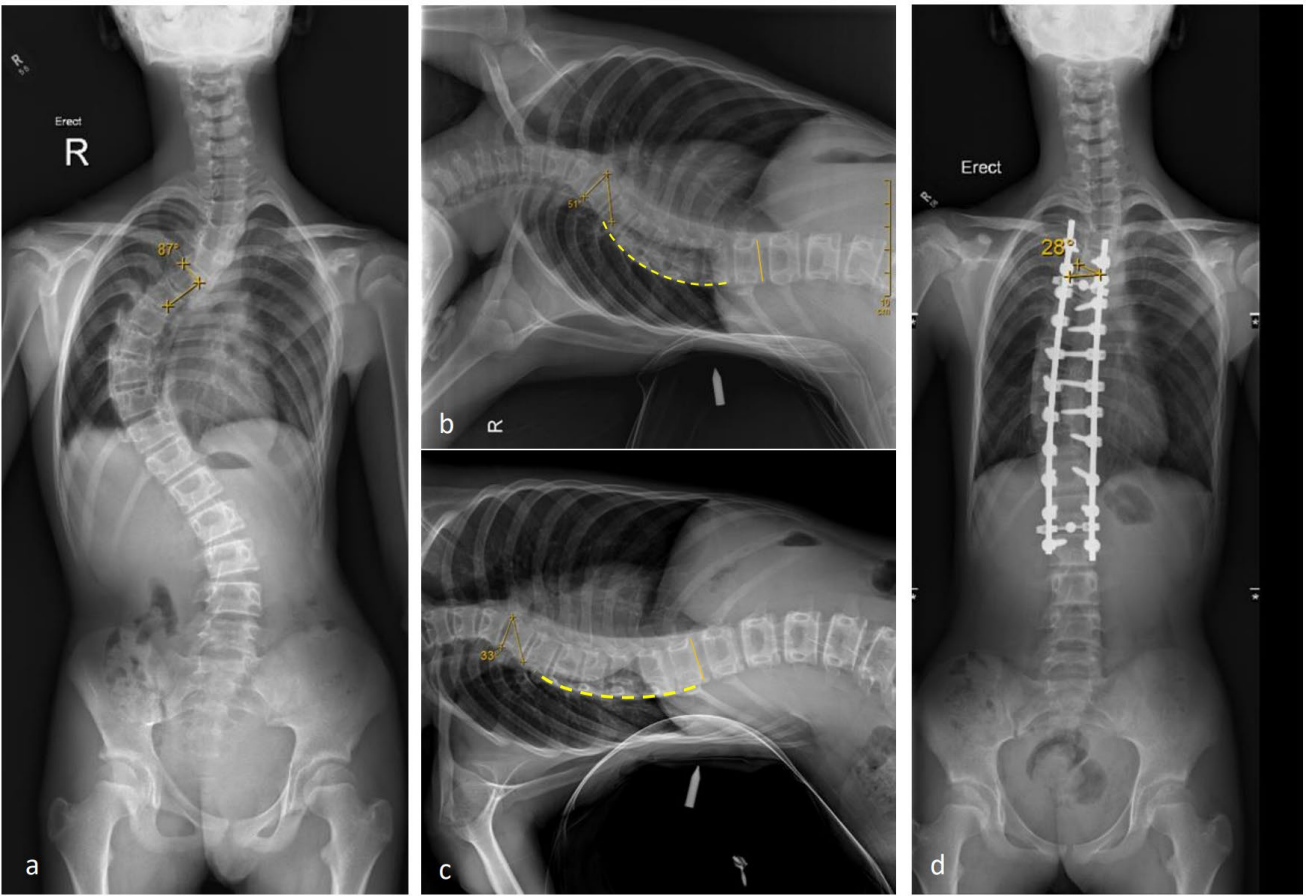
Radiographs of case 1. **a** Preoperatively taken whole-spine standing anteroposterior radiograph depicting a right thoracic curve with an 87° from T5–L1. **b** Pre-SSE fulcrum-bending radiograph showing a right thoracic curve with a 51° from T5–T11. **c** Post-SSE fulcrum-bending radiograph displaying a right thoracic curve with a 33° from T5–T11. **d** Whole-spine, standing anteroposterior radiograph, taken at postoperative day 5, displaying postoperative correction with a 28° from T5–L1

In this study, 21 patients in the prospective cohort exhibited rigid scoliosis. A 5-day inpatient SSE program was implemented to improve each patient’s preoperative spinal flexibility. The results indicated an improvement in preoperative spinal flexibility in patients with rigid thoracic scoliosis. After the 5-day inpatient SSE program, subsequent fulcrum-bending radiography revealed Cobb angles of 23 ± 7° at T4/5 to T11 (spinal flexibility: 66%), indicating a 16° improvement compared with the results of pre-SSE fulcrum-bending radiography (pre-SSE fulcrum bending: 40 ± 9°, spinal flexibility: 39%) in the SSE group. For instance, a 12-year-old girl with right thoracic AIS (Cobb angle = 87° at T5-12, Fig. 2a) experienced a rapid progression of 20° within 1 year. Spinal fusion was recommended to halt this progression. Pre-SSE fulcrum-bending radiography revealed a 22° reduction in Cobb angle (37% correction in the initial curve magnitude, Fig. 2b), which was further enhanced by an additional 18° reduction after the 5-day SSE program (Fig. 2c). Postoperative standing radiography revealed a 28° curve magnitude, which is 5° less than the Cobb angle identified using post-SSE fulcrum-bending radiography (Fig. 2d). Taken together, these findings suggest that our 5-day inpatient SSE program can improve the preoperative spinal flexibility of patients with rigid thoracic spinal scoliosis. This program can also improve respiratory function. Severe thoracic spinal scoliosis is more likely to cause respiratory dysfunction, thereby increasing the risk of postoperative complications such as pulmonary infection. Therefore, in addition to improving preoperative flexibility, our 5-day inpatient SSE program can potentially improve preoperative pulmonary function, offering clinical value in reducing the risk of postoperative respiratory complications.

ERAS is a multidisciplinary approach focused on improving perioperative outcomes through the use of subspecialty-specific, evidence-based protocols in surgical care [27]. For patients with AIS, the focus of preoperative physical therapy is enhancing truncal muscle strength and respiratory function, which may in turn shorten hospital stays and reduce readmission rates. In this study, we routinely provided perioperative exercise instruction for patients undergoing spinal surgery to facilitate recovery and mitigate the risk of postoperative complications. For patients with rigid scoliosis in the prospective cohort, a favorable intraoperative curve correction of 40 ± 7° was achieved without neurological complications. In addition to these benefits, the SSE program effectively improved the patients’ preoperative spinal flexibility. Specifically, the patients’ initial flexibility, which represented a 39% correction, increased to 66% after the SSE program. Without this improvement in preoperative spinal flexibility, the predicted range of postoperative correction would have been only 45° to 50°, necessitating more extensive release and a longer operative time to achieve optimal curve correction intraoperatively [6]. There were three cases that undertook Ponte osteotomy in this study; hence, we could not conclude that SSE eliminated the stronger surgical techniques. It requests prospective cohort study to address this research question. Notably, the simplicity of preoperative exercises, absence of extended hospitalization, and lack of complications contributed to maximum treatment satisfaction, as reported by the patients. In addition, the SSE group reported less pain than did the non-SSE groups after surgery, suggesting that the preoperative exercises alleviated muscle tightness and thereby aided in the management of postoperative pain. Taken together, our findings support the potential clinical value of establishing SSE guidelines for perioperative care in patients with severe scoliosis. Large-scale studies are required to explore the effect of SSE on preoperative spinal flexibility in patients with severe scoliosis across different curve magnitudes.

The main limitation of this study was its small sample size. Although we examined 65 patients with thoracic scoliosis, we could not confirm whether SSE improves spinal flexibility in all patients with varying curve patterns or determine whether an extended duration of SSE would have had additional effects on spinal flexibility and surgical outcomes. Nevertheless, our power analysis confirmed that our findings were sufficiently significant to suggest that our 5-day inpatient SSE program can improve preoperative spinal flexibility. Other limitations included the lack of subject blinding and the absence of standing radiographs after SSE. The patients and physiotherapists were aware of the SSE program, which was designed to maximize the effects of SSE on spinal flexibility. We did not use post-SSE standing radiography because of the potential for unnecessary radiation exposure and the spinal flexibility can be adequately evaluated through fulcrum-bending rather than standing radiography. In addition, the reliability of the pain outcomes in the retrospective cohort was compromised by the variability observed in pain management preferences, measurement inconsistencies, and the effect of surgical techniques. These factors should be addressed by employing a prospective study design. Moreover, the choice of the administrative model for the preoperative SSE program, that is, whether a 5-day exercise regimen with an inpatient setting or a longer-duration exercise with an outpatient framework, warrants discussion in future studies. In this study, we opted for an inpatient model because of the superior learning effects of supervised exercises [10]. Nevertheless, hospitalization arrangements differ globally, and outpatient models or home exercises may be a feasible option for patients awaiting surgery. Furthermore, a cost-effectiveness study is required to evaluate the financial and clinical feasibility of preoperative SSE. To the best of our knowledge, this is the first study to implement intensive SSE to improve preoperative flexibility and facilitate curve correction. Given that the effectiveness of SSE relies on patient cooperation and the quality of exercise, we recommend supervised training for patients with rigid scoliosis who are preparing for surgery. Overall, this study lays the foundation for future research into the effects of SSE on preoperative spinal flexibility in patients with stiff scoliosis.

## Conclusion

A novel non-operative optimization technique with 5-day inpatient SSE has been adopted to improve preoperative spinal flexibility. This study suggested that 5-day inpatient SSE improved the patient’s preoperative Cobb angle with fulcrum-bending radiograph and facilitated curve correction.

## Data Availability

Raw data analyzed in this study are included in the supplementary file 1, and patients’ radiographic films (SSE group) are included in the supplementary file 2.

## Availability of data and materials

Data have open access after publication.
